# Molecular Mechanisms and Therapeutic Strategies in Heart Failure Due to Dystrophin Deficiency: A Comprehensive Review

**DOI:** 10.31083/RCM46389

**Published:** 2026-03-16

**Authors:** Wanqian Yu, Wang Wang, Fan Luo, Yuanbin Zhao, Qinghua Wu, Ping Li

**Affiliations:** ^1^Department of Cardiovascular Medicine, The Second Affiliated Hospital, Jiangxi Medical College, Nanchang University, 330006 Nanchang, Jiangxi, China; ^2^Department of Gastroenterology, Jiangxi Provincial Hospital of Traditional Chinese Medicine, 330006 Nanchang, Jiangxi, China; ^3^Department of Cardiovascular Medicine, Jingdezhen First People’s Hospital, 333000 Jingdezhen, Jiangxi, China

**Keywords:** dystrophin, Duchenne muscular dystrophy, heart failure, gene therapy

## Abstract

Dystrophin deficiency is the core pathological feature of Duchenne muscular dystrophy (DMD) and Becker muscular dystrophy (BMD). Indeed, a deficiency in dystrophin results in the progressive degeneration of skeletal muscle and severely compromises the structure and function of cardiomyocytes, eventually leading to dilated cardiomyopathy and heart failure. Thus, this review provides an in-depth analysis of the molecular mechanisms underlying dystrophin-deficient cardiomyopathy, including membrane instability, calcium dysregulation, mitochondrial dysfunction, and fibrosis. The role of inflammatory responses in disease progression is also discussed. In addition, we evaluate current and emerging therapeutic strategies, including gene therapy, pharmacological interventions, and regenerative medicine approaches, and highlight recent preclinical and clinical trial data. Finally, we explore future directions in precision medicine, novel biomarkers for early detection, and combination treatment regimens, to provide a comprehensive resource for clinicians and researchers working in this challenging field.

## 1. Introduction

Dystrophin is a crucial protein that maintains the structural integrity of muscle cells by linking the cytoskeleton to the extracellular matrix [1]. Its absence or deficiency, as seen in Duchenne muscular dystrophy (DMD) and Becker muscular dystrophy (BMD), leads to the progressive degeneration of both skeletal and cardiac muscles [2]. While DMD is more commonly associated with skeletal muscle weakness, cardiac involvement becomes a major concern as the disease progresses, particularly in the form of dystrophin-deficient cardiomyopathy (DDCM), which is responsible for much of the morbidity and mortality in these patients [3].

Cardiac complications, such as dilated cardiomyopathy (DCM), often emerge in DMD and BMD patients in their teens or early twenties, leading to reduced ejection fraction, arrhythmias, and ultimately heart failure [4]. This makes the heart a critical organ in dystrophinopathies, necessitating a deeper understanding of the molecular mechanisms underlying cardiac dysfunction. Key factors contributing to cardiac dysfunction include membrane instability, calcium dysregulation, mitochondrial dysfunction, and fibrosis [5]. In the absence of dystrophin, mechanical stress leads to membrane damage and calcium influx, triggering cellular damage, inflammation, and fibrosis, all of which contribute to a decline in cardiac function.

Despite recent advances in understanding the molecular basis of DCM, effective therapies for this condition in DMD and BMD patients are limited. However, emerging treatments like gene therapy, stem cell-based therapies, and targeted pharmacological interventions hold great promise for slowing or halting disease progression [6]. This review aims to provide a concise synthesis of our current understanding of the molecular mechanisms underlying DDCM. We also explore the potential of emerging therapeutic strategies for DCM.

## 2. Molecular Mechanisms Underlying Dystrophin-Deficient Cardiomyopathy

### 2.1 Membrane Instability and Structural Disruption

Dystrophin deficiency in cardiomyocytes severely compromises the integrity of the sarcolemma, which is the cell membrane of cardiac muscle cells [7]. The absence of this key structural protein prevents proper linking of the actin cytoskeleton to the extracellular matrix (ECM), leading to significant vulnerability of the heart muscle to mechanical stress during contraction. In DMD patients, repetitive mechanical injury to the sarcolemma causes localized membrane tears that allow the influx of calcium and other ions into the cell, disrupting cellular homeostasis [7]. This disruption is associated with immediate damage and also triggers a cascade of events that contribute to cell death and muscle degeneration. Additionally, the compromised ability of the sarcolemma to repair itself exacerbates this process, making the myocardium prone to progressive dysfunction [8].

Recent studies have highlighted that dystrophin-deficient hearts exhibit a distinct pattern of membrane damage and cell death compared to hearts with traditional forms of heart failure [7, 9]. This specific pattern underscores the uniqueness of dystrophinopathies and suggests that targeted therapies should consider the mechanistic role of membrane instability as a critical therapeutic target.

### 2.2 Calcium Dysregulation and Mitochondrial Dysfunction

Under normal physiological conditions, calcium is tightly regulated in heart cells to maintain proper contraction and relaxation cycles [10]. However, in the absence of dystrophin, the sarcolemma’s fragility leads to calcium influx, causing pathological elevation of intracellular calcium levels. This abnormal calcium influx disrupts normal cellular signaling and triggers a cascade of downstream events that compromise cellular function.

Intracellular calcium overload activates proteolytic enzymes, such as calpains, which degrade structural proteins, leading to the breakdown of essential cellular structures. This contributes to the deterioration of sarcomeres, the contractile units of muscle fibers, and ultimately leads to myocardial dysfunction [11]. Mitochondria, which are responsible for generating energy in the form of Adenosine Triphosphate (ATP), are overwhelmed by calcium and begin to produce reactive oxygen species (ROS). ROS damage cellular components, including lipids, proteins, and DNA, contributing to the overall oxidative stress within cells [12]. This vicious cycle of calcium overload, mitochondrial dysfunction, and oxidative stress exacerbates the damage to cardiomyocytes and accelerates heart failure. Importantly, mitochondrial dysfunction further impairs the ability of the sarcolemma to repair itself, as mitochondria play a role in providing the energy required for membrane stabilization [13].

The intertwined relationship between calcium dysregulation and mitochondrial damage suggests that therapies aimed at normalizing calcium levels or protecting mitochondria could provide substantial benefits for patients with dystrophin-deficient heart failure.

### 2.3 Inflammatory Responses and Fibrotic Remodeling

Dystrophin-deficient hearts undergo significant inflammatory responses due to the constant cycle of muscle damage and repair [14]. In the early stages of injury, dying cardiomyocytes release pro-inflammatory cytokines such as Tumor Necrosis Factor-alpha (TNF-α) and Interleukin-6 (IL-6), which attract immune cells to the site of damage [15]. These immune cells, including macrophages and neutrophils, exacerbate the inflammatory state by secreting additional cytokines and matrix-degrading enzymes. Over time, the persistent inflammation leads to activation of cardiac fibroblasts, which deposit ECM proteins to form fibrosis [16]. Fibrotic tissue disrupts the normal heart architecture, impairing electrical conduction and increasing the risk of arrhythmias.

Persistent cytokine signaling (e.g., TNF-α, IL-6) potentiates sarcoplasmic reticulum calcium leak and channel dysfunction [17, 18], while Transforming Growth Factor-beta (TGF-β)–driven fibrosis increases electrical and mechanical heterogeneity, both of which feed back to exacerbate calcium dysregulation and injury to the sarcolemma [19]. The progression of fibrosis is closely linked to the exacerbation of heart failure symptoms in dystrophin-deficient patients. Interventions that target the inflammatory response, such as the use of TGF-β inhibitors, have shown promise in reducing myocardial fibrosis and improving overall cardiac function [20].

### 2.4 Metabolic Alterations and Additional Molecular Insights

Recent findings have highlighted the significant role of metabolic dysfunction in DDCM. In addition to calcium dysregulation and mitochondrial dysfunction, alterations in lipid metabolism and reduced mitochondrial fatty acid oxidation contribute significantly to disease progression [21]. These metabolic changes increase oxidative stress and disrupt energy production, further impairing mitochondrial function. The failure to efficiently utilize fatty acids for energy exacerbates the pathological processes already present due to mitochondrial dysfunction. Additionally, altered nitric oxide signaling has been observed in dystrophin-deficient hearts, which may compromise vascular function and myocardial perfusion [22]. Abnormal nitric oxide metabolism, combined with mitochondrial dysfunction, creates a vicious cycle that accelerates cardiac dysfunction.

The contribution of these metabolic changes to disease progression underscores the importance of considering metabolic therapy as a potential intervention for dystrophinopathies. The investigation of pharmacological agents that could restore normal metabolic processes in cardiomyocytes is a promising direction for future research.

### 2.5 Dystrophin and the Cardiac Cytoskeleton

The cardiac cytoskeleton plays an essential role in maintaining the shape and mechanical integrity of cardiomyocytes [23]. Dystrophin is an integral component of this structure, functioning to link the actin cytoskeleton to the ECM through the dystrophin-glycoprotein complex. This structural framework is compromised in the absence of dystrophin, making the myocardium more susceptible to mechanical stress [24]. The structural integrity of cardiomyocytes is crucial for efficient contractile function, and its loss contributes significantly to the progression of cardiomyopathy in DMD and BMD patients.

Disruption of the cytoskeletal network in dystrophin-deficient hearts has been shown to lead to a series of mechanical and biochemical abnormalities, including altered sarcomere structure and impaired force transmission during contraction [25]. The inability to withstand mechanical stress causes progressive myocardial damage, resulting in further degeneration of cardiomyocytes. Moreover, the loss of dystrophin also impairs the mechanical coupling of cardiomyocytes, which affects the contractile force and overall efficiency of the heart [26].

This phenomenon further emphasizes the need to develop therapeutic strategies that restore the structural integrity of the cardiac cell membrane. Strategies that focus on strengthening the cytoskeletal network or targeting the dystrophin-glycoprotein complex could potentially halt the progression of cardiomyopathy in dystrophinopathies [27].

### 2.6 Autophagy and the Role of Cellular Repair

Autophagy is the process by which cells degrade and recycle their components. It plays a crucial role in maintaining cellular homeostasis and in the cell response to stress [28]. Autophagic processes are often impaired in dystrophin-deficient hearts, leading to the accumulation of dysfunctional mitochondria and other damaged cellular components. The failure of cellular repair mechanisms compounds the oxidative stress and mitochondrial dysfunction already present, further accelerating cardiomyocyte death and myocardial degeneration. Studies have shown that enhancing autophagy may have beneficial effects on cellular survival, tissue repair, and mitochondrial function in dystrophin-deficient hearts [29].

The targeting of autophagy-related pathways could provide a novel therapeutic angle. Recent research has indicated that pharmacological modulation of autophagy could improve cellular health by promoting the clearance of damaged cellular components, thus potentially mitigating the adverse effects of dystrophin deficiency [30].

### 2.7 Pathogenesis and Mechanistic Insights Into Dystrophin-Deficient Cardiomyopathy

**Fig. 1.**
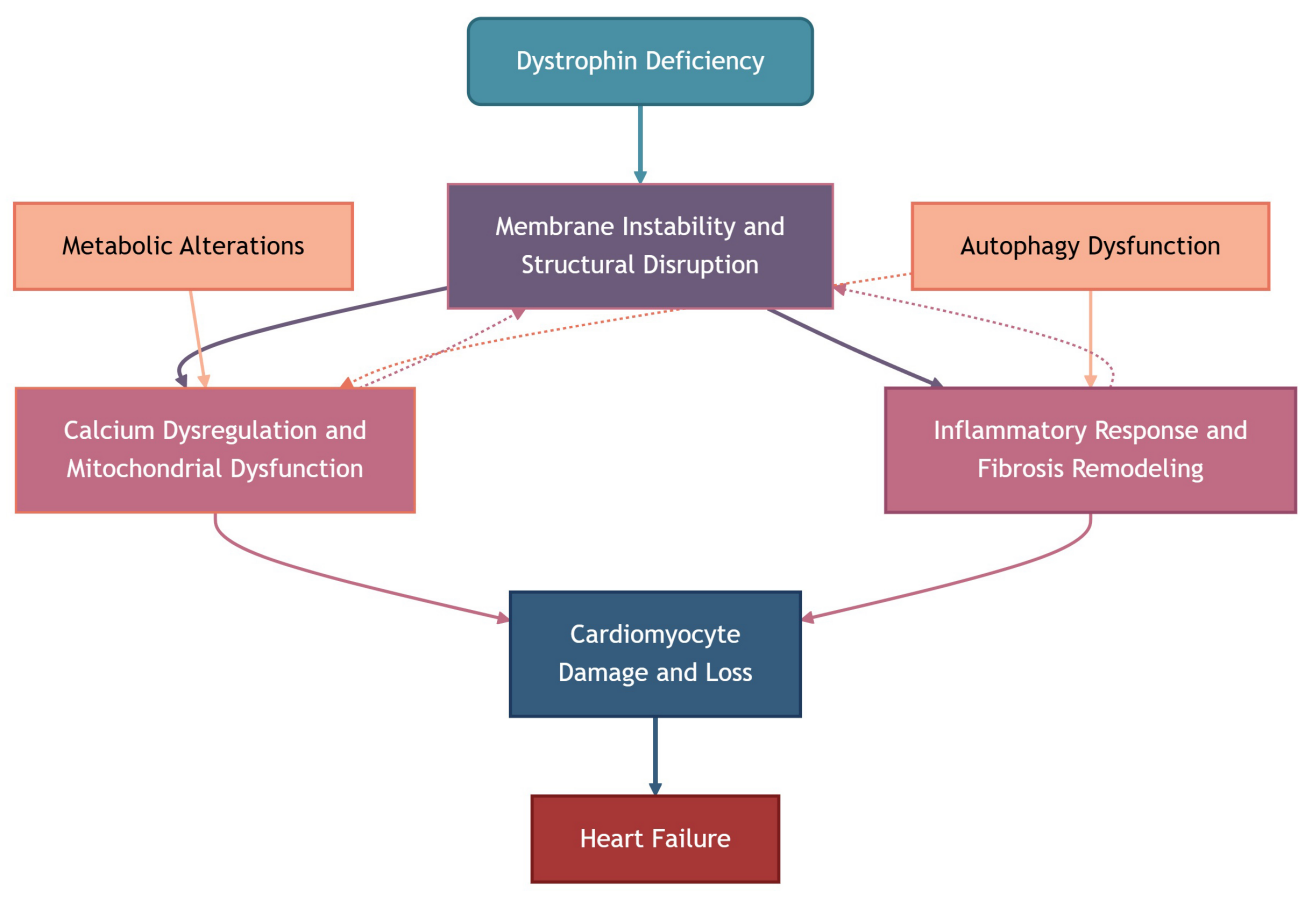
**Molecular mechanisms underlying dystrophin-deficient cardiomyopathy**. Solid arrows indicate direct causal relationships, while dashed arrows represent exacerbating or feedback effects.

The interplay between these pathological mechanisms ultimately leads to progressive cardiomyocyte loss, fibrosis, and heart failure in DDCM (Fig. 1). Understanding these interconnected pathways provides valuable insights into potential therapeutic targets. Approaches aimed at stabilizing the sarcolemma, restoring calcium homeostasis, reducing fibrosis, and improving metabolic function hold promise for mitigating disease progression.

## 3. Clinical Manifestations and Diagnostic Challenges

### 3.1 Progressive Decline in Cardiac Function

As previously described, cardiac dysfunction in patients with DMD and BMD typically begins to manifest in adolescence. The progressive nature of heart failure in these individuals often leads to significant delays in diagnosis, as skeletal muscle degeneration generally precedes cardiac symptoms [31]. However, the onset of cardiac symptoms is insidious and can be masked by the concurrent skeletal muscle weakness. In the early stages, left ventricular dilation and reduced ejection fraction are commonly observed with echocardiography [32].

Given the high variability in disease progression, clinical practitioners face challenges in predicting the exact timeline of heart failure in dystrophin-deficient patients. Early biomarkers, such as elevated levels of cardiac troponin I or other myocardial proteins, are critical for detecting subclinical cardiac dysfunction. Additionally, advanced imaging techniques like cardiac magnetic resonance (CMR) imaging, which offer more detailed insights into myocardial fibrosis and structure, have become essential tools for monitoring disease progression [33].

### 3.2 Arrhythmias and Sudden Cardiac Death

Arrhythmias are a major cause of mortality in dystrophin-deficient patients. Structural changes, including fibrosis and abnormal electrical conduction pathways, contribute to the development of arrhythmias, often leading to sudden cardiac death [34]. Prolonged exposure to arrhythmic events can significantly impair quality of life, especially in adolescent and young adult patients. To better manage these risks, continuous cardiac monitoring and proactive interventions are critical.

Greater awareness of the arrhythmogenic substrate in dystrophinopathies has prompted the exploration of new therapies aimed at stabilizing myocardial electrical activity [35]. The use of antiarrhythmic medications, implantable defibrillators, and pacemakers is becoming more common in clinical practice. Nevertheless, the benefits of these interventions are often limited, underscoring the need for therapies that target the underlying molecular causes of arrhythmia.

### 3.3 Emerging Diagnostic Tools and Biomarker Development

Traditional imaging modalities such as echocardiography and CMR imaging are useful in evaluating structural and functional abnormalities [36]. However, these tools often detect changes only after significant damage has occurred. To facilitate earlier intervention, research is increasingly focused on the development of sensitive biomarkers. Circulating biomarkers such as cardiac troponin I, various microRNAs (miRNAs), and other myocardial-specific proteins are under investigation as potential early indicators of cardiac involvement [37]. Table 1 (Ref. [38, 39, 40, 41, 42, 43, 44, 45]) provides a comprehensive overview of these emerging biomarkers and their detection methods, performance, and clinical significance.

**Table 1.** **Overview of emerging biomarkers for early detection of cardiac involvement in dystrophinopathies**.

Biomarker	Detection method	Performance	Clinical significance	Key references
Cardiac troponin I (high-sensitivity assays)	Serum/plasma	Quantitative sensitivity/specificity for DMD cardiac involvement are not uniformly reported; longitudinal hs-cTnI correlates with cardiac involvement and is recommended for a prospective study.	Candidate early myocardial injury marker in DMD/BMD; useful for longitudinal monitoring, but thresholds and prognostic cutoffs are still under study.	Spurney *et al*., 2021 [38]; Yamaguchi *et al*., 2022 [42]
miR-1, miR-133a/b	Plasma/serum qPCR or sequencing	Multiple DMD cohorts report high AUCs distinguishing DMD vs controls (ROC/AUC reported in some studies; e.g., many myomiRs show AUCs >0.80 in cohort analyses). Exact sensitivity/specificity varies by cohort and assay.	Robust circulating markers of muscle injury; some (miR-1, miR-133) associate with cardiac involvement in cohort studies—promising for early detection but need cardiac-focused prospective validation.	Zaharieva *et al*., 2013 [43]; Li *et al*., 2014 [44]; Meng *et al*., 2022 [39]
miR-208a/b, miR-499 (cardiac myomiRs)	Plasma/serum qPCR	Reported dysregulation in DMD; in non-DMD cardiology literature, these have high specificity for cardiac injury. Cohort studies in DMD report correlations with fibrosis/CMR changes.	More cardiac-specific than skeletal myomiRs; candidate markers for myocardial injury/fibrosis in dystrophinopathies.	Jeanson-Leh *et al*., 2014 [40]; Liu *et al*., 2015 [41]
Exosome/EV-associated miRNAs and proteins	Plasma/serum exosome profiling (RNA-seq, MS)	Emerging studies report altered EV-cargo in DMD; sensitivity/specificity not yet standardized across platforms.	Exosomal cargo may improve tissue-specificity and stability versus free miRNA; technical standardization and large prospective validation are needed.	Yedigaryan and Sampaolesi, 2023 [45]

AUC, area under the ROC curve; BMD, Becker muscular dystrophy; CMR, cardiac magnetic resonance; cTnI, cardiac troponin I; DMD, Duchenne muscular dystrophy; EV, extracellular vesicle; hs-cTnI, high-sensitivity cardiac troponin I; miR-1, MicroRNA 1; miR-133a/b, MicroRNA 133a/b; miR-208a/b, MicroRNA 208a/b; miR-499, MicroRNA 499; miRNA, microRNA; myomiR, muscle-enriched microRNA; MS, mass spectrometry; qPCR, quantitative polymerase chain reaction; RNA-seq, RNA sequencing; ROC, receiver operating characteristic.

Among these, the detection of cardiac troponin I with high-sensitivity assays is a plausible early indicator of myocardial injury and has shown longitudinal associations with cardiac involvement in dystrophinopathies [38]. Muscle-specific miRNAs released into the circulation following myocyte damage, such as miR-1 and miR-133a/b, have also shown promise. Significantly elevated serum levels of miR-1, miR-133a, and miR-133b were found in DMD patients compared to healthy controls, and these levels were higher in patients with documented cardiac involvement [39]. Although much of this work pertains to skeletal muscle pathology, several studies have suggested potential applicability to cardiac manifestations of dystrophinopathy. Another review noted that miR-1 and miR-133 were widely investigated in cardiovascular diseases as biomarkers of myocardial injury and fibrosis [46], although prospective, cardiac-endpoint–anchored validation and standardized clinical cutoff levels are still lacking [39]. Cardiac-biased myomiRs (miR-208a/b, miR-499) show greater cardiac specificity and have been linked to fibrosis or CMR changes in smaller cohorts. However, their lower circulating levels and the heterogeneity of assays limit their immediate clinical translation [40, 41]. Extracellular vesicles (EV) such as exosomes can carry miRNAs (including miR-1, miR-133) and may provide enhanced specificity for cardiac tissue injury. A recent review of exosomal miRNAs in cardiovascular disease highlights their value as non-invasive biomarkers and identifies miR-1 and miR-133 among those most frequently detected [47]. Nonetheless, while these biomarkers are promising, their specificity for cardiac vs skeletal muscle pathology in DMD/BMD patients remains to be firmly established, and large-scale clinical validation is still lacking [48].

Advances in molecular imaging techniques that target specific cellular and metabolic processes may also provide earlier detection of pathological changes.

### 3.4 Gender Differences in Disease Progression

While DMD and BMD are X-linked recessive disorders, gender differences have been observed in disease presentation [49]. For example, female carriers of DMD may exhibit variable degrees of cardiac involvement, with some experiencing progressive heart failure despite their relatively normal skeletal muscle function [50]. This is particularly evident in female carriers with skewed X-inactivation patterns, where the defective X chromosome is preferentially silenced, leading to lower levels of dystrophin expression in cardiac tissue.

In addition to the traditional male phenotype, female carriers of dystrophinopathies may show a more subtle onset of cardiomyopathy, often going undiagnosed until later in life. The occurrence of heart failure in these female carriers has attracted attention, as it suggests the need for more tailored screening and monitoring strategies [51]. Research is increasingly focused on understanding the underlying genetic and epigenetic factors that contribute to the variability in disease progression. This could lead to more personalized approaches in the diagnosis and management of DDCM [52].

### 3.5 Advanced Imaging Techniques for Early Diagnosis

Recent advancements in non-invasive imaging technologies are helping to detect early changes in the myocardium that may not yet be clinically apparent with conventional methods. For example, CMR imaging can identify myocardial fibrosis, a hallmark of heart failure, long before the onset of overt symptoms [53]. This ability to detect early pathological changes opens the door for interventions that could slow or halt the progression of heart failure.

Similarly, speckle tracking echocardiography is an emerging technique that assesses myocardial strain and can detect subclinical myocardial dysfunction, even in the absence of significant structural changes [54]. This technique holds promise as a diagnostic tool for the early identification of cardiac involvement in patients with dystrophinopathies. As these advanced imaging methods become more widely available, they may revolutionize the timing of interventions, allowing clinicians to initiate therapies before the occurrence of irreversible damage [55].

## 4. Therapeutic Strategies

Effective therapeutic strategies are crucial for managing heart failure related to dystrophin deficiency. This section provides an overview of current and emerging approaches, including gene therapy, pharmacological interventions, and regenerative medicine. Table 2 presents a comprehensive summary of these strategies, detailing their mechanisms, current status, advantages, and challenges.

**Table 2.** **Therapeutic strategies for dystrophin-deficient cardiomyopathy**.

Therapeutic strategy	Mechanism of action	Key examples	Current status	Advantages	Challenges
Gene therapy	Partially restore dystrophin expression	Micro-dystrophin gene therapy	Clinical trial (Phase III)	Addresses root cause, reduces membrane instability	Immune responses to viral vectors, optimal delivery route
Anti-fibrotic agents	modulate the fibrotic response	TGF-β inhibitors	Preclinical	Reduces fibrosis, improves cardiac function	Lack of clinical research
Calcium-regulating agents	Restore calcium homeostasis, modulate calcium flux	Ryanodine receptor stabilizers	Preclinical	Stabilizes Ca^2+^ levels, protects cardiomyocytes	Lack of clinical research
Regenerative medicine	Repair damaged myocardium, tissue regeneration	iPSC-derived cardiomyocytes	Preclinical	Integrate with host tissue, improve contractile function	Survival, maturation, Immune responses, Safety concerns
Combination therapies	Target multiple pathological pathways simultaneously	Gene therapy and pharmacological agents	Preclinical	Synergistic benefits, comprehensive approach	Requires carefully designed clinical trials, complexity
Gene editing	Directly correct genetic mutations for dystrophin deficiency	CRISPR/Cas9	Preclinical	Potential for long-term, curative treatment	Minimizing off-target effects, long-term stability, and ethical concerns

iPSC, induced pluripotent stem cells; CRISPR/Cas9, Clustered Regularly Interspaced Short Palindromic Repeats/CRISPR-associated protein 9; TGF-β, Transforming Growth Factor-beta.

### 4.1 Gene Therapy and Micro-Dystrophin Replacement

Gene therapy has emerged as one of the most promising approaches for treating dystrophin deficiency. Recent advances in viral vector technologies have enabled the delivery of micro-dystrophin constructs that can partially restore dystrophin expression in affected tissues [56]. Micro-dystrophin gene therapy has been shown to reduce membrane instability, stabilize calcium levels, and prevent further cardiomyocyte death in a preclinical model [57].

Building on encouraging preclinical results, clinical trials of gene therapy for DMD-related heart failure have advanced considerably, including the EMBARK Phase 3 randomized trial (NCT05096221) [57]. Continued advances in gene delivery systems and micro-dystrophin therapy hold great promise as components of a comprehensive treatment strategy. Nonetheless, significant challenges remain, including immune responses to viral vectors, optimal delivery route, and the long-term stability of dystrophin expression [58].

### 4.2 Pharmacological Interventions: Anti-Fibrotic and Calcium-Regulating Agents

While current pharmacological therapies for heart failure (e.g., angiotensin-converting enzyme [ACE] inhibitors, beta-blockers) have shown some efficacy in slowing disease progression, they do not target the primary molecular defects [59]. Recent research has focused on agents that modulate the fibrotic response and restore calcium homeostasis. TGF-β inhibitors, for example, have been shown to reduce myocardial fibrosis in preclinical models [60]. Similarly, the angiotensin receptor–neprilysin inhibitor Sacubitril/Valsartan has demonstrated potential not only in modulating neurohormonal pathways, but also in improving calcium handling within cardiomyocytes. the exploratory real-world study phase is currently completed, with improvements in ejection fraction and enhanced safety observed [61]. Recent pharmacological studies have also explored calcium channel blockers, which can potentially modulate calcium flux and reduce its toxic effects on cardiomyocytes. These agents act to stabilize calcium levels in the heart and protect it from the deleterious effects of calcium overload [62]. However, due to limitations in clinical translation, it remains only at the exploratory clinical stage [63].

In addition to calcium channel blockers, other therapeutic agents that target calcium handling, such as ryanodine receptor stabilizers currently in preclinical investigation, are being investigated. These drugs act on intracellular calcium release channels to prevent excessive calcium release from the sarcoplasmic reticulum, a major source of intracellular calcium overload [64, 65]. By stabilizing calcium homeostasis, these agents may offer a complementary treatment to gene therapy or stem cell-based therapies in dystrophin-deficient heart failure.

### 4.3 Stem Cell and Regenerative Medicine Approaches

Regenerative medicine holds promise for repairing damaged myocardium in dystrophin-deficient hearts. The use of induced pluripotent stem cells (iPSCs) to generate patient-specific cardiomyocytes is one of the most exciting developments in this field [66]. Experimental studies have shown that iPSC-derived cardiomyocytes can integrate with host tissue, improve contractile function, and even contribute to the regeneration of damaged myocardium [67]. However, significant challenges remain regarding the survival, maturation, and long-term functionality of these transplanted cells. Further research into optimizing cell delivery methods and ensuring proper integration with the host’s cardiac tissue is ongoing.

Translational challenges include:

• Cell survival and integration: The survival of transplanted cells within the damaged myocardium remains a major issue. Host immune responses may lead to cell rejection, and the harsh ischemic and inflammatory environment of the heart poses a challenge for cell integration. A recent study highlighted the need for optimized immunosuppressive strategies to reduce immune rejection and support graft persistence [68].

• Cell maturation: iPSC-derived cardiomyocytes do not always mature to a functional adult-like state, thus affecting their ability to contribute to long-term heart function. The development of strategies to promote proper maturation and integration into the host tissue is crucial. Current research has shown promise in using small molecules and engineered scaffolds to enhance cell maturation [69].

• Immune responses: Although iPSC-derived cells are patient-specific, immune activation still poses a risk, particularly in cases where non-autologous sources of stem cells are used, or if the cells undergo genetic modifications. The use of immunomodulatory agents or the development of immune-tolerant iPSCs may help to mitigate adverse responses [70].

• Safety concerns: Long-term safety remains a concern, particularly the risk of tumorigenicity from undifferentiated iPSCs. Effective strategies for controlling the differentiation of these cells and avoiding tumor formation are critical. Ongoing work in the field has focused on purifying differentiated cells and ensuring that only fully mature cardiomyocytes are transplanted [71].

While significant progress has been made in improving the delivery methods and optimizing cell survival, additional research is needed to address these challenges. Clinical trials must focus on ensuring the long-term efficacy and safety of these therapies, with special attention paid to minimizing immune responses and improving cell function. In combination with gene therapy or pharmacological agents, stem cell therapies may ultimately hold the key to reversing or halting the progression of heart failure in dystrophin-deficient patients.

### 4.4 Combination and Multi-Targeted Therapies

Given the multifactorial nature of DDCM, a single therapeutic modality may not be sufficient to halt disease progression. Combination therapies that target multiple pathological pathways simultaneously are being explored [72]. For example, integrating gene therapy with pharmacological agents that reduce fibrosis and oxidative stress could offer synergistic benefits. Similarly, combining regenerative medicine approaches with anti-inflammatory treatments might enhance the survival and integration of transplanted cells. This type of strategy remains in the experimental stage, and rigorous clinical trials are needed to assess its effectiveness and safety [73, 74].

### 4.5 Gene Editing Approaches for Dystrophin Restoration

Gene editing technologies, particularly Clustered Regularly Interspaced Short Palindromic Repeats/CRISPR-associated protein 9 (CRISPR/Cas9), have emerged as powerful tools for directly correcting the mutations responsible for dystrophin deficiency. Recent advances in genome editing have demonstrated that dystrophin expression can be restored in animal models of DMD through precise edits of the dystrophin gene [75]. These promising results have prompted ongoing efforts to adapt CRISPR technology for clinical applications, with the potential to correct genetic mutations in human patients [76]. While still in the preclinical stages, the use of CRISPR to correct dystrophin mutations holds significant promise for providing a long-term, potentially curative treatment for DMD and BMD patients [77].

However, several translational challenges still hinder the widespread clinical application of these therapies:

• Delivery systems: One of the key challenges in genome editing is the efficient delivery of CRISPR/Cas9 components to the target cells. Non-viral delivery systems, such as nanoparticles, are promising but often suffer from low efficiency and poor targeting. Viral vectors, while more efficient, carry risks of immune responses and off-target effects [78].

• Immune responses: The use of CRISPR/Cas9 may trigger immune reactions, particularly when viral vectors are used for delivery. These immune responses can reduce the effectiveness of treatment and increase the risk of side effects. A key area of ongoing research is the development of CRISPR systems that minimize immune activation [79].

• Off-target effects: Although the precision of CRISPR/Cas9 technology has improved significantly, off-target mutations remain a concern. These unintended genetic changes could potentially lead to adverse outcomes, such as the activation of oncogenes or the disruption of other important genes. Researchers are now focused on improving the specificity of CRISPR systems by using more refined versions of Cas9, such as high-fidelity Cas9 variants [80].

• Long-term stability: Another challenge is ensuring the long-term stability of dystrophin expression. Even if initial therapeutic effects are achieved, maintaining stable gene expression over time in a patient’s heart and muscle tissue is critical. Concerns remain about the durability of CRISPR-based edits, particularly after the treated cells divide [81].

• Ethical concerns: Beyond the technical challenges, gene editing for therapeutic purposes raises ethical questions, particularly regarding germline editing or the potential for unintended consequences in the genetic code. Regulatory frameworks will need to address these concerns as clinical trials move forward [82].

Despite these challenges, CRISPR-based therapies hold significant promise for treating dystrophinopathies. As research progresses, attention must be paid to minimizing off-target effects and optimizing delivery systems to ensure safe and effective gene editing in human patients [83]. Once such issues are addressed, gene editing could offer a long-term, potentially curative solution for DMD and BMD patients.

## 5. Preclinical Models and Translational Research

### 5.1 Animal Models and Their Role in Therapy Development

Animal models have played a crucial role in advancing our understanding of dystrophin-deficient cardiomyopathy. The mdx mouse is a widely used model of DMD that recapitulates many features of the human disease, including progressive skeletal muscle weakness and cardiac dysfunction [84]. This model has been instrumental in testing new therapies, ranging from gene editing to pharmacological intervention [1].

However, despite their value, animal models often fail to fully replicate the complexity of the human condition, particularly with regard to disease heterogeneity and long-term therapeutic outcomes. Consequently, there is an ongoing need for more sophisticated models that better mirror human disease and provide more predictive data for clinical trials. The development of large animal models, such as pigs and non-human primates, could provide more accurate representations of the human cardiovascular system and enable better testing of new treatments.

### 5.2 Advances in Genome Editing Technologies

The advent of genome editing tools, particularly CRISPR/Cas9, has opened new avenues for directly correcting the genetic mutations responsible for dystrophin deficiency. Recent studies have demonstrated that CRISPR-mediated approaches can restore dystrophin expression in animal models, leading to improvements in both skeletal and cardiac muscle function [75]. Although still in the experimental phase, these technologies represent a promising strategy for future therapeutic interventions that could potentially offer a permanent cure [85].

### 5.3 Translational Challenges and Future Research Directions

While preclinical models have provided invaluable insights, several challenges remain in translating these findings to the clinic. Significant hurdles remain in terms of the heterogeneity of the patient population, issues related to immune responses, and the need for long-term efficacy data, as detailed in Section 4 [86]. Future research must focus on bridging the gap between bench and bedside through well-designed clinical trials, the development of robust biomarkers for early detection, and the refinement of therapeutic delivery systems.

## 6. Emerging Technologies and Novel Therapeutic Targets

### 6.1 Precision Medicine and Genomic Profiling

In recent years, the concept of precision medicine has gained significant momentum in the treatment of complex diseases, including dystrophin-deficient cardiomyopathy. Advances in genomic profiling technologies, such as next-generation sequencing, have led to a deeper understanding of the genetic mutations that contribute to dystrophinopathies [87]. These technologies can also identify genetic variants that modulate disease severity, thus providing valuable insights into how individual patients may respond to different therapeutic strategies.

Precision medicine in the context of dystrophinopathies goes beyond simply correcting the underlying dystrophin deficiency. By considering individual genetic factors, clinicians can tailor interventions to minimize adverse side effects and maximize therapeutic efficacy [88]. For example, by identifying specific mutations or epigenetic modifications that influence the course of disease, therapies could be personalized to more effectively target both the underlying genetic cause and downstream pathological processes such as fibrosis, inflammation, and mitochondrial dysfunction.

Furthermore, the incorporation of precision medicine can enhance the development of novel therapeutic strategies. Pharmacogenomic approaches, which consider how genetic variations affect drug responses, can optimize the use of existing medications and improve outcomes in patients with DMD and BMD [89]. Tailored therapies could also involve the use of gene editing technologies or RNA-based therapies that are customized according to the specific genetic profile of each patient, ultimately improving their clinical management and outcomes.

### 6.2 Immunomodulatory Approaches

Chronic inflammation plays a pivotal role in the progression of DDCM, contributing to both myocardial fibrosis and the ongoing cycle of muscle degeneration. Therefore, immunomodulatory therapies that target inflammatory pathways have gained considerable attention as potential therapeutic options for dystrophinopathies [90]. The goal of immunomodulation in this context is to reduce chronic inflammation without compromising the body’s ability to fight infections and other diseases.

Several cytokines and immune signaling pathways, including TNF-α, IL-6, and TGF-β, have been identified as key mediators of inflammation in dystrophinopathies. Inhibiting these cytokines or their receptors may reduce inflammation and fibrosis, potentially slowing the progression of heart failure in dystrophin-deficient patients [91]. For example, therapies that target TGF-β signaling have shown promise in preclinical studies by reducing myocardial fibrosis and improving cardiac function [92].

Regulatory T cell therapies, which aim to enhance the body’s natural anti-inflammatory response, are also being investigated as a potential treatment strategy to mitigate inflammation without exacerbating immune suppression. When combined with other therapeutic modalities such as gene therapy or stem cell therapy, these approaches could help to control the inflammatory process that drives myocardial damage in dystrophinopathies.

## 7. Clinical Trial Updates and Future Perspectives

### 7.1 Recent Clinical Trials

Several clinical trials are currently underway to evaluate novel therapies for DDCM. Early-phase trials of micro-dystrophin gene therapy have reported encouraging preliminary results, with improvements in cardiac function and safety profiles that support further investigation [57]. Similarly, trials investigating the efficacy of Sacubitril/Valsartan and TGF-β inhibitors in reducing myocardial fibrosis have shown promising results that could pave the way for more targeted heart failure therapies in DMD and BMD patients [72].

### 7.2 Challenges in Clinical Translation

Despite these advances, significant challenges remain in the clinical translation of experimental therapies. One of the major hurdles is the variability in disease progression and treatment response among patients [93]. In addition, ensuring the long-term safety and efficacy of gene and cell-based therapies remains a concern. Multidisciplinary collaboration and well-designed, large-scale clinical trials are essential to overcome these obstacles and bring effective therapies to the clinic [94, 95].

### 7.3 Future Directions in Research and Treatment

The future management of DDCM lies in the integration of precision medicine, advanced biomarker development, and combination therapeutic strategies. Ongoing research should focus on:

• Refining gene editing and gene therapy techniques to maximize efficacy and minimize immune reactions.

• Expanding the use of iPSC-derived cardiomyocytes and improving their long-term integration into host tissue.

• Developing robust, non-invasive biomarkers for early detection and real-time monitoring of disease progression.

• Designing combination therapies that simultaneously target multiple pathological pathways, such as inflammation, fibrosis, and metabolic dysfunction.

• Conducting comprehensive longitudinal studies to evaluate the long-term outcomes of novel therapies.

## 8. Conclusion

Dystrophin deficiency initiates a cascade of molecular and cellular events that culminate in the development of cardiomyopathy and heart failure in patients with DMD and BMD. The multifactorial nature of this disease, encompassing membrane instability, calcium dysregulation, mitochondrial dysfunction, inflammatory responses, and metabolic alterations, necessitates a comprehensive, multi-targeted therapeutic approach. Emerging treatments, including gene therapy, pharmacological agents that target fibrosis and calcium handling, and regenerative medicine, have shown promise in preclinical and early clinical trials. However, challenges related to early diagnosis, treatment variability, and long-term efficacy remain.

Future research efforts should focus on integrating precision medicine approaches, developing novel biomarkers, and exploring combination therapies that address the complex interplay of pathogenic mechanisms. With continued advances in genomic technologies, immunomodulatory strategies, and regenerative medicine, there is optimism that more effective treatments will become available to improve the quality of life and survival of patients with DDCM.

## Abbreviations

ACE, Angiotensin-Converting Enzyme; ATP, Adenosine Triphosphate; AUC, Area Under the ROC Curve; BMD, Becker muscular dystrophy; CMR, Cardiac Magnetic Resonance; CRISPR/Cas9, Clustered Regularly Interspaced Short Palindromic Repeats/CRISPR-associated protein 9; cTnI, Cardiac Troponin I; DCM, dilated cardiomyopathy; DDCM, dystrophin-deficient cardiomyopathy; DMD, Duchenne muscular dystrophy; ECM, Extracellular Matrix; EV, Extracellular Vesicle; hs-cTnI, High-Sensitivity Cardiac Troponin I; IL-6, Interleukin-6; iPSCs, Induced Pluripotent Stem Cells; miR-1, MicroRNA 1; miR 133a/b, MicroRNA 133a/b; miR-208a/b, MicroRNA 208a/b; miR-499, MicroRNA 499; miRNAs, MicroRNAs; MS, Mass Spectrometry; myomiR, Muscle-Enriched MicroRNA; qPCR, Quantitative Polymerase Chain Reaction; RNA-seq, RNA Sequencing; ROC, Receiver Operating Characteristic; ROS, Reactive Oxygen Species; TGF-β, Transforming Growth Factor-beta; TNF-α, Tumor Necrosis Factor-alpha.

## References

[1] Hoffman EP, Brown RH, Kunkel LM (1987). Dystrophin: the protein product of the Duchenne muscular dystrophy locus. *Cell*.

[2] McNally EM, Kaltman JR, Benson DW, Canter CE, Cripe LH, Duan D (2015). Contemporary cardiac issues in Duchenne muscular dystrophy. Working Group of the National Heart, Lung, and Blood Institute in collaboration with Parent Project Muscular Dystrophy. *Circulation*.

[3] Moretti A, Liguori S, Paoletta M, Gimigliano F, Iolascon G (2022). Effectiveness of Neridronate in the Management of Bone Loss in Patients with Duchenne Muscular Dystrophy: Results from a Pilot Study. *Advances in Therapy*.

[4] Dai Y, Wang Y, Fan Y, Han B (2025). Genotype-phenotype insights of pediatric dilated cardiomyopathy. *Frontiers in Pediatrics*.

[5] Ziemba M, Barkhouse M, Uaesoontrachoon K, Giri M, Hathout Y, Dang UJ (2021). Biomarker-focused multi-drug combination therapy and repurposing trial in mdx mice. *PLoS ONE*.

[6] Rieger AC, Myerburg RJ, Florea V, Tompkins BA, Natsumeda M, Premer C (2019). Genetic determinants of responsiveness to mesenchymal stem cell injections in non-ischemic dilated cardiomyopathy. *eBioMedicine*.

[7] Fullenkamp DE, Willis AB, Curtin JL, Amaral AP, Dittloff KT, Harris SI (2024). Physiological stress improves stem cell modeling of dystrophic cardiomyopathy. *Disease Models & Mechanisms*.

[8] Hueneke R, Adenwala A, Mellor RL, Seidman JG, Seidman CE, Nerbonne JM (2017). Early remodeling of repolarizing K+ currents in the αMHC403⁣/+ mouse model of familial hypertrophic cardiomyopathy. *Journal of Molecular and Cellular Cardiology*.

[9] Haddad CN, Ali S, Stephanou D, Assakura MS, Sahagian L, Trogkanis E (2023). Pharmacological management of dilated cardiomyopathy in Duchenne muscular dystrophy: A systematic review. *Hellenic Journal of Cardiology*.

[10] Wen Q, Zhang R, Ye K, Yang J, Shi H, Liu Z (2024). Empagliflozin rescues pro-arrhythmic and Ca2+ homeostatic effects of transverse aortic constriction in intact murine hearts. *Scientific Reports*.

[11] Zhang H, Guo H, Han F, Zheng Y (2025). Regulatory mechanisms of m6A methylation in dilated cardiomyopathy. *American Journal of Translational Research*.

[12] Wang L, Li W, Liu Y, Dilixiati A, Chang Z, Liang Y (2025). Spermidine Supplementation Effectively Improves the Quality of Mouse Oocytes After Vitrification Freezing. *Antioxidants*.

[13] Vila MC, Rayavarapu S, Hogarth MW, Van der Meulen JH, Horn A, Defour A (2017). Mitochondria mediate cell membrane repair and contribute to Duchenne muscular dystrophy. *Cell Death and Differentiation*.

[14] Law ML, Prins KW, Olander ME, Metzger JM (2018). Exacerbation of dystrophic cardiomyopathy by phospholamban deficiency mediated chronically increased cardiac Ca2+ cycling in vivo. *American Journal of Physiology. Heart and Circulatory Physiology*.

[15] Zhuang YT, Xu DY, Wang GY, Sun JL, Huang Y, Wang SZ (2017). IL-6 induced lncRNA MALAT1 enhances TNF-α expression in LPS-induced septic cardiomyocytes via activation of SAA3. *European Review for Medical and Pharmacological Sciences*.

[16] Zuo GF, Wang LG, Huang L, Ren YF, Ge Z, Hu ZY (2024). TAX1BP1 downregulation by STAT3 in cardiac fibroblasts contributes to diabetes-induced heart failure with preserved ejection fraction. *Biochimica et Biophysica Acta. Molecular Basis of Disease*.

[17] Fauconnier J, Meli AC, Thireau J, Roberge S, Shan J, Sassi Y (2011). Ryanodine receptor leak mediated by caspase-8 activation leads to left ventricular injury after myocardial ischemia-reperfusion. *Proceedings of the National Academy of Sciences of the United States of America*.

[18] Keefe JA, Aguilar-Sanchez Y, Navarro-Garcia JA, Ong I, Li L, Paasche A (2025). Macrophage-mediated IL-6 signaling drives ryanodine receptor-2 calcium leak in postoperative atrial fibrillation. *The Journal of Clinical Investigation*.

[19] Frangogiannis NG (2022). Transforming growth factor-β in myocardial disease. *Nature Reviews. Cardiology*.

[20] Feng R, Liu H, Chen Y (2025). Baricitinib represses the myocardial fibrosis via blocking JAK/STAT and TGF-β1 pathways in vivo and in vitro. *BMC Cardiovascular Disorders*.

[21] Guo Z, Shi Y, Jiang B, Peng X, Zhang L, Tu C (2023). Psoraleae Fructus Ethanol Extract Induced Hepatotoxicity via Impaired Lipid Metabolism Caused by Disruption of Fatty Acid β-Oxidation. *Oxidative Medicine and Cellular Longevity*.

[22] Lopez JR, Kolster J, Zhang R, Adams J (2017). Increased constitutive nitric oxide production by whole body periodic acceleration ameliorates alterations in cardiomyocytes associated with utrophin/dystrophin deficiency. *Journal of Molecular and Cellular Cardiology*.

[23] West G, Sedighi S, Agnetti G, Taimen P (2023). Intermediate filaments in the heart: The dynamic duo of desmin and lamins orchestrates mechanical force transmission. *Current Opinion in Cell Biology*.

[24] Meyers TA, Heitzman JA, Krebsbach AM, Aufdembrink LM, Hughes R, Bartolomucci A (2019). Acute AT1R blockade prevents isoproterenol-induced injury in mdx hearts. *Journal of Molecular and Cellular Cardiology*.

[25] Dahiya S, Givvimani S, Bhatnagar S, Qipshidze N, Tyagi SC, Kumar A (2011). Osteopontin-stimulated expression of matrix metalloproteinase-9 causes cardiomyopathy in the mdx model of Duchenne muscular dystrophy. *Journal of Immunology*.

[26] Pereyra AS, Fernandez RF, Amorese A, Castro JN, Lin CT, Spangenburg EE (2024). Loss of mitochondria long-chain fatty acid oxidation impairs skeletal muscle contractility by disrupting myofibril structure and calcium homeostasis. *Molecular Metabolism*.

[27] Murphy S, Dowling P, Zweyer M, Mundegar RR, Henry M, Meleady P (2016). Proteomic analysis of dystrophin deficiency and associated changes in the aged mdx-4cv heart model of dystrophinopathy-related cardiomyopathy. *Journal of Proteomics*.

[28] Liu Q, Chen Y, Zhou L, Chen H, Zhou Z (2022). From Intestinal Epithelial Homeostasis to Colorectal Cancer: Autophagy Regulation in Cellular Stress. *Antioxidants*.

[29] Chen X, Sun T, Qi Y, Zhu B, Li L, Yu J (2025). Paeoniflorin ameliorates reperfusion injury in H9C2 cells through SIRT1-PINK1/parkin-mediated mitochondrial autophagy. *Molecular Immunology*.

[30] Sebori R, Kuno A, Hosoda R, Hayashi T, Horio Y (2018). Resveratrol Decreases Oxidative Stress by Restoring Mitophagy and Improves the Pathophysiology of Dystrophin-Deficient mdx Mice. *Oxidative Medicine and Cellular Longevity*.

[31] Alizadeh M, Ghasemi H, Bazhan D, Mohammadi Bolbanabad N, Rahdan F, Arianfar N (2025). MicroRNAs in disease States. *Clinica Chimica Acta; International Journal of Clinical Chemistry*.

[32] Sumi K, Masuda T, Kondo H, Obayashi K, Takeuchi Y, Harada T (2025). Cardiac involvement and anti-striational antibodies in immune-mediated necrotizing myopathy. *Journal of the Neurological Sciences*.

[33] Yang D, Jiang Y, Qian H, Liu X, Mi L (2021). Silencing Cardiac Troponin I-Interacting Kinase Reduces Lipopolysaccharide-Induced Sepsis-Induced Myocardial Dysfunction in Rat by Regulating Apoptosis-Related Proteins. *BioMed Research International*.

[34] Sauer J, Marksteiner J, Lilliu E, Hackl B, Todt H, Kubista H (2024). Empagliflozin treatment rescues abnormally reduced Na+ currents in ventricular cardiomyocytes from dystrophin-deficient mdx mice. *American Journal of Physiology. Heart and Circulatory Physiology*.

[35] Papa AA, D’Ambrosio P, Petillo R, Palladino A, Politano L (2017). Heart transplantation in patients with dystrophinopathic cardiomyopathy: Review of the literature and personal series. *Intractable & Rare Diseases Research*.

[36] Sewanan LR, Di Tullio MR, Laine AF, D’Souza B, Leb J, Mironov A (2023). Absence of long-term structural and functional cardiac abnormalities on multimodality imaging in a multi-ethnic group of COVID-19 survivors from the early stage of the pandemic. *European Heart Journal. Imaging Methods and Practice*.

[37] Drakenberg A, Sundqvist AS, Fridlund B, Ericsson E (2024). On a healing journey together and apart: A Swedish critical incident technique study on family involvement from a patient perspective in relation to elective open-heart surgery. *Scandinavian Journal of Caring Sciences*.

[38] Spurney CF, Ascheim D, Charnas L, Cripe L, Hor K, King N (2021). Current state of cardiac troponin testing in Duchenne muscular dystrophy cardiomyopathy: review and recommendations from the Parent Project Muscular Dystrophy expert panel. *Open Heart*.

[39] Meng Q, Zhang J, Zhong J, Zeng D, Lan D (2022). Novel miRNA Biomarkers for Patients With Duchenne Muscular Dystrophy. *Frontiers in Neurology*.

[40] Jeanson-Leh L, Lameth J, Krimi S, Buisset J, Amor F, Le Guiner C (2014). Serum profiling identifies novel muscle miRNA and cardiomyopathy-related miRNA biomarkers in Golden Retriever muscular dystrophy dogs and Duchenne muscular dystrophy patients. *The American Journal of Pathology*.

[41] Liu X, Fan Z, Zhao T, Cao W, Zhang L, Li H (2015). Plasma miR-1, miR-208, miR-499 as potential predictive biomarkers for acute myocardial infarction: An independent study of Han population. *Experimental Gerontology*.

[42] Yamaguchi H, Awano H, Yamamoto T, Nambu Y, Iijima K (2022). Serum cardiac troponin I is a candidate biomarker for cardiomyopathy in Duchenne and Becker muscular dystrophies. *Muscle & Nerve*.

[43] Zaharieva IT, Calissano M, Scoto M, Preston M, Cirak S, Feng L (2013). Dystromirs as serum biomarkers for monitoring the disease severity in Duchenne muscular Dystrophy. *PLoS One*.

[44] Li X, Li Y, Zhao L, Zhang D, Yao X, Zhang H (2014). Circulating Muscle-specific miRNAs in Duchenne Muscular Dystrophy Patients. *Molecular Therapy. Nucleic Acids*.

[45] Yedigaryan L, Sampaolesi M (2023). Extracellular vesicles and Duchenne muscular dystrophy pathology: Modulators of disease progression. *Frontiers in Physiology*.

[46] Song Z, Gao R, Yan B (2020). Potential roles of microRNA-1 and microRNA-133 in cardiovascular disease. *Reviews in Cardiovascular Medicine*.

[47] Qingpiao S, Yi Z (2025). Exosomal miRNAs: A New Frontier in Cardiovascular Disease Diagnosis and Treatment. *Journal of Cardiovascular Translational Research*.

[48] Fortunato F, Ferlini A (2023). Biomarkers in Duchenne Muscular Dystrophy: Current Status and Future Directions. *Journal of Neuromuscular Diseases*.

[49] Medori R, Brooke MH, Waterston RH (1989). Genetic abnormalities in Duchenne and Becker dystrophies: clinical correlations. *Neurology*.

[50] Bamaga AK, Alghamdi F, Alshaikh N, Altwaijri W, Bashiri FA, Hundallah K (2021). Consensus Statement on the Management of Duchenne Muscular Dystrophy in Saudi Arabia During the Coronavirus Disease 2019 Pandemic. *Frontiers in Pediatrics*.

[51] Lupu M, Pintilie IM, Teleanu RI, Marin GG, Vladâcenco OA, Severin EM (2025). Early Cardiac Dysfunction in Duchenne Muscular Dystrophy: A Case Report and Literature Update. *International Journal of Molecular Sciences*.

[52] Viggiano E, Picillo E, Cirillo A, Politano L (2013). Comparison of X-chromosome inactivation in Duchenne muscle/myocardium-manifesting carriers, non-manifesting carriers and related daughters. *Clinical Genetics*.

[53] Carbucicchio C, Guarracini F, Schiavone M, Gasperetti A, Conte E, Preda A (2024). Preprocedural imaging with cardiac computed tomography for endo-epicardial ventricular tachycardia ablation. *Heart Rhythm*.

[54] Yilmaz A, Gdynia HJ, Baccouche H, Mahrholdt H, Meinhardt G, Basso C (2008). Cardiac involvement in patients with Becker muscular dystrophy: new diagnostic and pathophysiological insights by a CMR approach. *Journal of Cardiovascular Magnetic Resonance*.

[55] Yildirim M, Salbach C, Reich C, Pribe-Wolferts R, Milles BR, Täger T (2024). Improved diagnostic performance of high-sensitivity cardiac troponins in muscle dystrophies using comprehensive definition criteria for cardiac involvement: A longitudinal study on 35 patients. *European Journal of Neurology*.

[56] Duan D (2018). Systemic AAV Micro-dystrophin Gene Therapy for Duchenne Muscular Dystrophy. *Molecular Therapy: The Journal of The American Society of Gene Therapy*.

[57] Mendell JR, Muntoni F, McDonald CM, Mercuri EM, Ciafaloni E, Komaki H (2025). AAV gene therapy for Duchenne muscular dystrophy: the EMBARK phase 3 randomized trial. *Nature Medicine*.

[58] Tasfaout H, Halbert CL, McMillen TS, Allen JM, Reyes TR, Flint GV (2024). Split intein-mediated protein trans-splicing to express large dystrophins. *Nature*.

[59] Bremner SB, Mandrycky CJ, Leonard A, Padgett RM, Levinson AR, Rehn ES (2022). Full-length dystrophin deficiency leads to contractile and calcium transient defects in human engineered heart tissues. *Journal of Tissue Engineering*.

[60] Wu H, Li GN, Xie J, Li R, Chen QH, Chen JZ (2016). Resveratrol ameliorates myocardial fibrosis by inhibiting ROS/ERK/TGF-β/periostin pathway in STZ-induced diabetic mice. *BMC Cardiovascular Disorders*.

[61] Arcudi A, Di Francesco M, Rodolico D, D’Amario D (2022). Angiotensin receptor-neprilysin inhibitor in symptomatic patients with Duchenne dilated cardiomyopathy: A primetime. *ESC Heart Failure*.

[62] Sun QA, Grimmett ZW, Hess DT, Perez LG, Qian Z, Chaube R (2024). Physiological role for S-nitrosylation of RyR1 in skeletal muscle function and development. *Biochemical and Biophysical Research Communications*.

[63] Pernice W, Beckmann R, Ketelsen UP, Frey M, Schmidt-Redemann B, Haap KP (1988). A double-blind placebo controlled trial of diltiazem in Duchenne dystrophy. *Klinische Wochenschrift*.

[64] Kaplan AD, Boyman L, Ward CW, Lederer WJ, Greiser M (2024). Ryanodine receptor stabilization therapy suppresses Ca2+- based arrhythmias in a novel model of metabolic HFpEF. *Journal of Molecular and Cellular Cardiology*.

[65] Vincenti M, Farah C, Amedro P, Scheuermann V, Lacampagne A, Cazorla O (2022). Early Myocardial Dysfunction and Benefits of Cardiac Treatment in Young X-Linked Duchenne Muscular Dystrophy Mice. *Cardiovascular Drugs and Therapy*.

[66] Zhang A, Liu Y, Pan J, Pontanari F, Chia-Hao Chang A, Wang H (2023). Delivery of mitochondria confers cardioprotection through mitochondria replenishment and metabolic compliance. *Molecular Therapy*.

[67] Barrett P, Louie KW, Dupont JB, Mack DL, Maves L (2024). Uncovering the Embryonic Origins of Duchenne Muscular Dystrophy. *WIREs Mechanisms of Disease*.

[68] Ito E, Kawamura A, Kawamura T, Takeda M, Harada A, Mochizuki-Oda N (2023). Establishment of a protocol to administer immunosuppressive drugs for iPS cell-derived cardiomyocyte patch transplantation in a rat myocardial infarction model. *Scientific reports*.

[69] Iwoń Z, Krogulec E, Tarnowska I, Łopianiak I, Wojasiński M, Dobrzyń A (2024). Maturation of human cardiomyocytes derived from induced pluripotent stem cells (iPSC-CMs) on polycaprolactone and polyurethane nanofibrous mats. *Scientific Reports*.

[70] Murata K, Ikegawa M, Minatoya K, Masumoto H (2020). Strategies for immune regulation in iPS cell-based cardiac regenerative medicine. *Inflammation and Regeneration*.

[71] Zhong C, Liu M, Pan X, Zhu H (2022). Tumorigenicity risk of iPSCs in vivo: nip it in the bud. *Precision Clinical Medicine*.

[72] Verhaart IEC, Aartsma-Rus A (2019). Therapeutic developments for Duchenne muscular dystrophy. *Nature Reviews. Neurology*.

[73] Wang W, Tayier B, Guan L, Yan F, Mu Y (2022). Pre-transplantation of Bone Marrow Mesenchymal Stem Cells Amplifies the Therapeutic Effect of Ultrasound-Targeted Microbubble Destruction-Mediated Localized Combined Gene Therapy in Post-Myocardial Infarction Heart Failure Rats. *Ultrasound in Medicine & Biology*.

[74] Gandhi S, Sweeney HL, Hart CC, Han R, Perry CGR (2024). Cardiomyopathy in Duchenne Muscular Dystrophy and the Potential for Mitochondrial Therapeutics to Improve Treatment Response. *Cells*.

[75] Long C, Amoasii L, Mireault AA, McAnally JR, Li H, Sanchez-Ortiz E (2016). Postnatal genome editing partially restores dystrophin expression in a mouse model of muscular dystrophy. *Science*.

[76] Amoasii L, Long C, Li H, Mireault AA, Shelton JM, Sanchez-Ortiz E (2017). Single-cut genome editing restores dystrophin expression in a new mouse model of muscular dystrophy. *Science Translational Medicine*.

[77] Andrysiak K, Machaj G, Priesmann D, Woźnicka O, Martyniak A, Ylla G (2024). Dysregulated iron homeostasis in dystrophin-deficient cardiomyocytes: correction by gene editing and pharmacological treatment. *Cardiovascular Research*.

[78] Huang J, Zhou Y, Li J, Lu A, Liang C (2022). CRISPR/Cas systems: Delivery and application in gene therapy. *Frontiers in Bioengineering and Biotechnology*.

[79] Legere NJ, Hinson JT (2024). Emerging CRISPR Therapies for Precision Gene Editing and Modulation in the Cardiovascular Clinic. *Current Cardiology Reports*.

[80] Bonowicz K, Jerka D, Piekarska K, Olagbaju J, Stapleton L, Shobowale M (2025). CRISPR-Cas9 in Cardiovascular Medicine: Unlocking New Potential for Treatment. *Cells*.

[81] Schreurs J, Sacchetto C, Colpaert RMW, Vitiello L, Rampazzo A, Calore M (2021). Recent Advances in CRISPR/Cas9-Based Genome Editing Tools for Cardiac Diseases. *International Journal of Molecular Sciences*.

[82] Grisorio L, Bongianino R, Gianeselli M, Priori SG (2024). Gene therapy for cardiac diseases: methods, challenges, and future directions. *Cardiovascular Research*.

[83] Xu L, Zhang C, Li H, Wang P, Gao Y, Mokadam NA (2021). Efficient precise in vivo base editing in adult dystrophic mice. *Nature Communications*.

[84] Bostick B, Shin JH, Yue Y, Duan D (2011). AAV-microdystrophin therapy improves cardiac performance in aged female mdx mice. *Molecular Therapy*.

[85] Nelson CE, Hakim CH, Ousterout DG, Thakore PI, Moreb EA, Castellanos Rivera RM (2016). In vivo genome editing improves muscle function in a mouse model of Duchenne muscular dystrophy. *Science*.

[86] Kodippili K, Hakim CH, Pan X, Yang HT, Yue Y, Zhang Y (2018). Dual AAV Gene Therapy for Duchenne Muscular Dystrophy with a 7-kb Mini-Dystrophin Gene in the Canine Model. *Human Gene Therapy*.

[87] Fratter C, Dalgleish R, Allen SK, Santos R, Abbs S, Tuffery-Giraud S (2020). EMQN best practice guidelines for genetic testing in dystrophinopathies. *European Journal of Human Genetics*.

[88] Aartsma-Rus A, Krieg AM (2017). FDA Approves Eteplirsen for Duchenne Muscular Dystrophy: The Next Chapter in the Eteplirsen Saga. *Nucleic Acid Therapeutics*.

[89] Landfeldt E, Aleman A, Abner S, Zhang R, Werner C, Tomazos I (2024). Factors Associated with Respiratory Health and Function in Duchenne Muscular Dystrophy: A Systematic Review and Evidence Grading. *Journal of Neuromuscular Diseases*.

[90] R A, Mohan S, Vellapandian C (2024). A Voyage on the Role of Nuclear Factor Kappa B (NF-kB) Signaling Pathway in Duchenne Muscular Dystrophy: An Inherited Muscle Disorder. *Cureus*.

[91] Townsend D, Turner I, Yasuda S, Martindale J, Davis J, Shillingford M (2010). Chronic administration of membrane sealant prevents severe cardiac injury and ventricular dilatation in dystrophic dogs. *The Journal of Clinical Investigation*.

[92] González-Herrera F, Catalán M, Anfossi R, Maya JD, Pedrozo Z, Díaz-Araya G (2023). SGK1 is necessary to FoxO3a negative regulation, oxidative stress and cardiac fibroblast activation induced by TGF-β1. *Cellular Signalling*.

[93] Johnstone VPA, Viola HM, Hool LC (2017). Dystrophic Cardiomyopathy-Potential Role of Calcium in Pathogenesis, Treatment and Novel Therapies. *Genes*.

[94] Mah ML, Cripe L, Slawinski MK, Al-Zaidy SA, Camino E, Lehman KJ (2020). Duchenne and Becker muscular dystrophy carriers: Evidence of cardiomyopathy by exercise and cardiac MRI testing. *International Journal of Cardiology*.

[95] Del Rio-Pertuz G, Morataya C, Parmar K, Dubay S, Argueta-Sosa E (2022). Dilated cardiomyopathy as the initial presentation of Becker muscular dystrophy: a systematic review of published cases. *Orphanet Journal of Rare Diseases*.

